# Rheological and Morphological Characterization of Asphalt Cement Hybrid-Modified with Waste-Derived Cellulose Fibers and Crumb Rubber

**DOI:** 10.3390/polym18192346

**Published:** 2026-09-26

**Authors:** Hümeyra Bolakar Tosun, Fatma Pınar Göksal

**Affiliations:** 1 Department of Civil Engineering, Aksaray University, Aksaray 68100, Türkiye; 2 Department of Industrial Engineering, Aksaray University, Aksaray 68100, Türkiye; fatmapinargoksal@aksaray.edu.tr

**Keywords:** asphalt binder, waste-derived filler, recycled cellulose fiber, crumb rubber, rheology, ageing, SEM, FTIR-ATR

## Abstract

Conventional bitumens may not adequately withstand increasing traffic loads and environmental stresses, necessitating the development of more durable and sustainable modified binder systems. This study investigated the rheological, workability, and morphological characteristics of a 50/70 penetration-grade asphalt binder hybrid-modified with waste-derived cellulose fibers and crumb rubber. Penetration, softening point, Brookfield viscosity, Dynamic Shear Rheometer (DSR), Scanning Electron Microscopy (SEM), and Fourier Transform Infrared Spectroscopy (FTIR) analyses were performed. The hybrid-modified binder exhibited a penetration value of 55 ± 2 (0.1 mm) and a softening point of 53 ± 1 °C. The G*/sinδ criterion was satisfied at 66 ± 1.5 °C, while the corresponding critical temperature reached 71.2 ± 1.2 °C after ageing, indicating enhanced high-temperature deformation resistance. However, the viscosity reached 2367 ± 45 mPa·s at 150 °C, indicating a trade-off between high-temperature performance and workability. SEM analysis revealed that modification transformed the relatively smooth and homogeneous surface of the base bitumen into a rougher, more heterogeneous, and structurally complex morphology containing partially embedded modifier-rich domains. Although localized agglomeration indicated that completely uniform dispersion was not achieved, the observed morphological changes supported the development of a physically reinforced binder structure. FTIR analysis showed that the principal chemical framework of the base bitumen was preserved following modification. Variations in the fingerprint region indicated contributions from the cellulose and crumb rubber phases, whereas the absence of distinct new absorption bands suggested that the modification mechanism was predominantly governed by physical blending, crumb-rubber swelling, mechanical interlocking, and weak secondary intermolecular interactions rather than the formation of new covalent bonds. Overall, the combined rheological, SEM, and FTIR findings demonstrate that waste-derived cellulose fibers and crumb rubber can improve asphalt binder performance while promoting waste valorization and supporting circular economy principles.

## 1. Introduction

The road construction sector is increasingly striving to reconcile infrastructure development with environmental sustainability, driven by global climate policies, resource scarcity, and the urgent need to reduce environmental degradation associated with conventional construction materials [1,2]. Among the most critical drivers of this transformation is the rapid growth of industrial and post-consumer waste, particularly end-of-life tires and discarded textile products, which pose severe environmental and waste management challenges worldwide [3]. These materials are highly resistant to natural degradation and occupy substantial landfill space, while uncontrolled disposal can lead to soil, water, and air contamination. Globally, approximately 1.5 billion tires reach the end of their useful life each year, yet a significant proportion is still disposed of without effective recycling or recovery processes, resulting in serious environmental burdens and resource losses [4]. In addition to its environmental implications, the road construction and asphalt industry represents a major global economic sector. According to the market analysis cited in Reference [5], the asphalt industry represents a substantial global economic sector. Its reported global market value of approximately USD 65–83 billion and projected growth, driven by infrastructure investment, urbanization, and increasing road maintenance requirements, reinforce the need to develop durable, cost-effective, and environmentally sustainable pavement materials.

Previous research has demonstrated that waste tires, particularly crumb rubber, can provide substantial engineering advantages when incorporated into cementitious and asphalt-based materials. These advantages include improved flexibility, enhanced resistance to water damage, and increased durability under repeated loading conditions [6,7,8]. In asphalt technology, crumb rubber modification has attracted extensive attention due to its ability to improve the viscoelastic properties of binders, particularly at high service temperatures. Experimental studies and comprehensive reviews have consistently shown that rubber-modified binders exhibit improved rutting resistance, enhanced fatigue life, and reduced susceptibility to thermal cracking, ultimately extending pavement service life and lowering maintenance costs [2,9,10,11,12]. From a sustainability perspective, the reuse of waste rubber also contributes to waste reduction and promotes circular material use in transportation infrastructure [12].

However, despite these engineering benefits, rubber modification is often associated with increased binder viscosity, which can negatively affect mixing, compaction, and processing conditions during pavement production [13,14]. Higher viscosity may require elevated mixing temperatures and increased energy consumption, potentially offsetting some of the environmental benefits. To address these limitations and develop more balanced and high-performance asphalt binder systems, researchers have increasingly explored the use of fiber-based additives in asphalt mixtures. Various types of fibers, including synthetic, natural, and recycled fibers, have demonstrated the ability to enhance mechanical performance, improve structural integrity, and delay crack propagation under repeated traffic loading [15,16,17]. Cellulose fibers have an elongated morphology and hydroxyl-rich surfaces that may promote physical adhesion and mechanical reinforcement within bitumen. Crumb-rubber particles have rough, irregular surfaces and can absorb lighter bitumen fractions and partially swell during mixing. These characteristics can restrict binder mobility and improve viscosity, elasticity, and resistance to permanent deformation.

Among these materials, cellulose fibers have gained particular attention due to their ability to stabilize asphalt mixtures, prevent binder drainage, and improve cohesion within the bituminous matrix. Cellulose fibers can absorb excess binder and create a reinforcing network structure within the mixture, improving resistance to deformation and crack initiation.

Cellulose and recycled textile fibers have been investigated as stabilizing and reinforcing additives in asphalt mixtures, particularly for controlling binder drainage and improving selected mechanical and durability-related properties [1,16,17]. In addition, fiber-reinforced asphalt mixtures often exhibit improved flexibility, reduced susceptibility to pothole formation, and enhanced long-term performance under varying environmental conditions.

While improvements have been reported using crumb rubber or cellulose fibers individually, combining multiple waste-derived additives may provide potentially complementary effects and help address different performance requirements within a single binder system. This hybrid modification approach represents a promising advancement in asphalt technology, as it allows the complementary properties of different waste materials to be exploited within a single binder system [17,18,19]. Rubber particles contribute primarily to elasticity, energy dissipation, and resistance to permanent deformation at high temperatures, whereas fiber reinforcement enhances structural stability, crack resistance, and flexibility, particularly at lower temperatures.

From an environmental perspective, the incorporation of waste rubber and textile-derived cellulose fibers into asphalt binders provides a sustainable alternative to direct disposal, reducing landfill pressure and minimizing environmental impacts associated with waste accumulation. This approach aligns with circular economy principles by transforming industrial and post-consumer waste into value-added engineering materials. Furthermore, the combined use of rubber and cellulose fibers not only enhances technical performance but also contributes to resource efficiency, waste valorization, and reduced environmental footprint in road construction. The selection of crumb rubber and cellulose fibers in this study is based on their complementary physicochemical and mechanical characteristics. Crumb rubber acts as an elastomeric phase that enhances energy dissipation within the bitumen matrix. In contrast, cellulose fibers function as a reinforcing structural network, improving binder stability, reducing drain-down, and enhancing load distribution within the composite system. It was hypothesized that cellulose fibers would provide physical reinforcement, while crumb rubber would improve the elastic response of the binder, thereby enhancing its overall rheological performance. The combined use of these materials was hypothesized to provide complementary effects; however, their elastic recovery and resistance to permanent deformation through swelling and interaction individual contributions and any potential effect, cannot be determined without corresponding single-modifier control binders.

This study investigates the rheological, workability, morphological, and physicochemical characteristics of a 50/70 penetration-grade asphalt binder hybrid-modified with 5 wt% waste-derived cellulose fibers and 7 wt% crumb rubber. Penetration, softening point, Brookfield viscosity, DSR, RTFOT, PAV, BBR, SEM, and FTIR analyses were performed to determine whether the complementary functions of the fibrous and elastomeric modifiers could improve high-temperature deformation resistance while maintaining acceptable workability and low-temperature performance. Previous studies have investigated crumb-rubber- and cellulose-modified asphalt systems separately, while related hybrid studies have mainly focused on asphalt-mixture performance [1,16,17,18]. In contrast, the present study evaluates recycled cotton-derived cellulose fibers and crumb rubber together directly at the binder scale. Its specific contribution is the integrated characterization of their combined effects through conventional tests, DSR, RTFOT, PAV, BBR, SEM, and FTIR-ATR analyses.

## 2. Materials and Methods

### 2.1. Materials and Material Characteristics

The sources, forms, preparation procedures, dosages, and available characteristics of the raw materials used in this study are summarized in Table 1.

A conventional 50/70 penetration-grade bitumen was selected as the base binder for this study due to its widespread use in flexible pavement applications and its well-documented rheological behavior under medium traffic and moderate climatic conditions. The base bitumen exhibited a Brookfield rotational viscosity of 320 mPa·s at 135 °C, indicating adequate pumpability and workability during mixing and compaction processes. At 60 °C, which represents typical high-service pavement temperatures, the binder demonstrated a complex shear modulus (G) of 1.20 kPa* and a phase angle (δ) of 86°, reflecting a predominantly viscous response with limited elastic recovery. These baseline rheological properties provided a reference framework for evaluating the performance enhancement achieved through sustainable material modification.

To improve both mechanical performance and environmental sustainability, a hybrid modification strategy was adopted incorporating recycled and waste-derived materials. The cellulose fibers were recovered from recycled cotton-based textile waste rather than obtained from standardized virgin cotton. Therefore, they exhibited heterogeneous dimensions and an irregular, elongated morphology. Before incorporation into the binder, the fibers were mechanically separated and cut into smaller pieces. The fiber-length distribution, diameter, and chemical purity were not measured separately; therefore, the variability of the recycled fibers is acknowledged as a limitation of this study. The prepared cellulose fibers were subsequently incorporated into the bituminous binder as a fibrous modifier. Because fiber aspect ratio and specific surface area were not measured quantitatively, no conclusions regarding these parameters were drawn. The fibrous structure was expected to contribute to binder stabilization and physical reinforcement; however, this interpretation was evaluated primarily through the rheological and morphological results obtained in the present study. The cellulose fibers were expected to act as dispersed physical inclusions that restrict binder mobility and locally reinforce the bituminous matrix.

In parallel, granulated waste tire rubber (crumb rubber) was incorporated as an elastomeric modifier. The crumb rubber was produced through ambient mechanical grinding of end-of-life tires, contributing to circular economy practices by diverting substantial volumes of non-biodegradable tire waste from landfills. The rubber particles exhibited elastic properties capable of absorbing stresses and enhancing strain recovery under cyclic loading. Upon interaction with the hot bitumen, partial swelling of rubber particles occurred due to the absorption of lighter aromatic fractions, leading to improved elasticity, increased complex modulus, and enhanced fatigue resistance. This elastomeric modification contributes significantly to cracking resistance at intermediate and low service temperatures. The final selected formulation consisted of 5 wt% recycled cellulose fibers and 7 wt% crumb rubber, while the remaining 88 wt% comprised base bitumen.

All modifier proportions reported in this study are expressed relative to the total mass of the prepared binder system. Therefore, the 5 wt% cellulose-fiber content should not be directly compared with the 0.2–0.5 wt% dosages commonly reported on the basis of the total asphalt-mixture mass. For example, in a mixture containing approximately 5 wt% binder, a cellulose dosage corresponding to 5 wt% of the binder would be equivalent to approximately 0.25 wt% of the total mixture. This illustrative conversion places the selected dosage within the range generally reported for mixture-level cellulose applications. The 5 wt% binder-based dosage was selected to provide sufficient fiber content for physical reinforcement while maintaining feasible mixing and handling conditions. The 7 wt% crumb-rubber content was selected based on literature-reported application ranges and preliminary mixing trials to improve elastic response and high-temperature deformation resistance while maintaining feasible mixing and processing conditions. Because a formal parametric optimization was not conducted, the selected composition should be regarded as an exploratory formulation rather than a fully optimized formulation.

The selection of this formulation was guided by preliminary evaluations and literature-reported dosage ranges. Previous studies [1,2,10,11,12,16,17] have reported that cellulose- and crumb-rubber-based modifiers can improve selected mechanical and rheological properties when appropriate dosages and processing conditions are used.

The selected hybrid formulation was designed to investigate the potentially complementary functions of cellulose fibers and crumb rubber. However, because corresponding cellulose-only and crumb-rubber-only binders were not included, the individual contribution of each modifier and the presence of a true synergistic effect could not be determined. From a sustainability perspective, the proposed formulation offers multiple environmental benefits:

#### 2.1.1. Waste Valorization

Textile waste, which constitutes a significant fraction of municipal solid waste globally, is repurposed as a functional reinforcement material. End-of-life tires are recycled into crumb rubber, mitigating long-term environmental hazards associated with tire stockpiling.

#### 2.1.2. Reduced Carbon Footprint

Incorporating recycled materials reduces the demand for virgin polymer modifiers and synthetic additives, thereby lowering embodied energy and greenhouse gas emissions associated with binder production. It should be noted that a comprehensive life cycle assessment (LCA) was not conducted within the scope of this study. The environmental benefits discussed are therefore based on established findings in the literature regarding the use of recycled materials in asphalt binder. Future research should include detailed LCA to quantitatively evaluate energy consumption, greenhouse gas emissions, and overall environmental impacts associated with the proposed modification approach.

#### 2.1.3. Extended Pavement Service Life

Enhanced rutting and fatigue resistance are expected to increase pavement durability, reducing maintenance frequency. Lower maintenance demand translates into reduced material consumption, energy use, traffic disruptions, and lifecycle emissions.

#### 2.1.4. Circular Economy Integration

The approach supports industrial symbiosis by connecting the textile, automotive, and construction sectors within a resource-efficient framework.

#### 2.1.5. Improved Thermal Performance

The elastomeric and fibrous network may improve temperature susceptibility behavior, contributing to climate-resilient infrastructure under increasing thermal variability associated with climate change.

Overall, the selected hybrid modification strategy not only targets rheological and mechanical performance enhancement but also strengthens the environmental and sustainability profile of asphalt pavements. By integrating waste-derived cellulose fibers and crumb rubber into conventional 50/70 penetration-grade bitumen, the study advances a multifunctional material design framework aligned with sustainable pavement engineering principles and circular resource management objectives.

### 2.2. Experimental Program

The conventional and rheological tests, including penetration, softening point, Brookfield viscosity, DSR, RTFOT, PAV, and BBR, were conducted at the accredited Bituminous Binders Laboratory of the General Directorate of Highways (KGM), Ankara, Türkiye. SEM and FTIR-ATR analyses were performed at the Scientific and Technological Application and Research Center of Aksaray University, Aksaray, Türkiye. The applicable standards, equipment manufacturers and models, experimental conditions, and measured parameters are summarized in Table 2.

The complex shear modulus (G*) was calculated as:

G* = √(G′^2^ + G″^2^)

where G′ is the storage modulus representing the elastic component, and G″ is the loss modulus representing the viscous component of the binder.

The phase angle (δ), which describes the viscoelastic behavior of the material, was defined as:

tan δ = G″/G′

where δ indicates the relative contribution of viscous and elastic responses.

For low-temperature performance, Bending Beam Rheometer (BBR) parameters were evaluated using creep stiffness (S) and m-value, defined as:

m = d(log S)/d(log t)

where S is the creep stiffness and t is the loading time. The m-value represents the rate of stress relaxation and is a key indicator of resistance to thermal cracking.

The BBR test temperature of −12 °C was selected in accordance with the Superpave low-temperature grading procedure. Under this procedure, testing at −12 °C corresponds to the evaluation of an approximate low-temperature performance grade of −22 °C because the BBR test temperature is 10 °C higher than the corresponding low pavement design temperature. The purpose of the test was to determine whether the modified binder satisfied the creep-stiffness and m-value requirements at this selected grade. Testing at additional lower temperatures was outside the scope of the present study.

All experimental measurements were conducted in at least three replicates, and the reported values represent the mean along with the corresponding standard deviation (±SD) to reflect measurement variability and experimental uncertainty.

### 2.3. Sample Preparation

Bitumen was heated gradually to 165 °C under continuous stirring (200 rpm). Waste-derived cellulose fibers and crumb rubber were incorporated at 5 wt% and 7 wt%, respectively, based on the total mass of the prepared binder system. Modified binders were stored at 135 °C for 24 h prior to testing.

The modified binders were prepared using a high-shear mixer under controlled temperature and mixing speed to ensure adequate dispersion of the modifiers within the bitumen. The mixing process was designed to minimize particle agglomeration and promote a homogeneous distribution of crumb rubber particles and cellulose fibers throughout the binder matrix.

### 2.4. SEM Characterization

SEM analyses were conducted at the Scientific and Technological Application and Research Center of Aksaray University using a [FEI, Quanta FEG 250] scanning electron microscope. The micrographs were obtained at magnifications of 2500× and 5000×. The morphology and dispersion characteristics of the unmodified and hybrid-modified asphalt binders were examined using Scanning Electron Microscopy (SEM). Before imaging, the samples were dried and mounted on aluminum stubs using conductive carbon tape. The analysis focused on the surface morphology of the modifiers, their dispersion within the bituminous matrix, particle-binder adhesion, possible mechanical constraint provided by the fibers, agglomeration, and void formation.

### 2.5. FTIR Characterization

FTIR-ATR analyses were conducted at the Scientific and Technological Application and Research Center of Aksaray University using a [Perkin Elmer, Spectrum Two] spectrometer. The spectra were recorded over the range of 4000–400 cm^−1^ at a resolution of 4 cm^−1^ for each spectrum. Fourier Transform Infrared Spectroscopy (FTIR) was performed to identify the principal functional groups and evaluate possible physicochemical interactions among the base bitumen, cellulose fibers, crumb rubber, and hybrid-modified binder. Background correction and baseline correction were applied before interpretation. The spectra were compared in terms of peak position, intensity, broadening, and the appearance or disappearance of characteristic absorption bands.

### 2.6. Data Reduction and Performance Criteria

Performance thresholds followed Superpave criteria: G*/sin δ ≥ 1 kPa for rutting resistance; G*sinδ ≤ 5000 kPa for intermediate-temperature fatigue resistance of the PAV-aged binder; BBR stiffness S ≤ 300 MPa and m-value ≥ 0.300 for thermal cracking resistance.

## 3. Results and Discussion

### 3.1. Fundamental Binder Properties

The hybrid-modified binder exhibited a penetration value of 55 (0.1 mm), indicating moderate stiffness and resistance to deformation. Its softening point was 53 °C, suggesting satisfactory high-temperature stability. Moreover, the modified binder satisfied the Superpave rutting criterion (G*/sinδ ≥ 1 kPa) at 66 °C, confirming its resistance to shear-induced permanent deformation (Table 3). Table 3 compares the experimentally determined properties of the original 50/70 bitumen and the hybrid-modified binder. Where experimental results were unavailable for the original binder, the applicable KGM specification limits according to TS EN 12591 are provided and clearly identified.

### 3.2. Aging Resistance and Rheological Performance

After RTFOT/PAV aging, the softening point increased by 11 °C, reflecting expected oxidative stiffening. The temperature at which G*/sin δ = 1 kPa rose to 71.2 °C, revealing greater stiffness and rutting resistance post-aging. PAV-aged G* sin δ = 5000 kPa occurred at 24.3 °C, indicating acceptable fatigue resistance. At −12 °C, the hybrid-modified binder exhibited a creep stiffness (S) of 185 ± 8 MPa and an m-value of 0.326 ± 0.006. Both parameters satisfied the Superpave requirements of S ≤ 300 MPa and m ≥ 0.300 (Table 4).

### 3.3. Viscosity and Workability

Brookfield viscosity at 150 °C was 2367 ± 45 mPa·s (torque = 94.7 ± 1.2%), necessitating higher mixing temperatures than conventional binders. A practical processing window emerged between 160 °C and 170 °C, balancing workability and stiffness. At 180 °C, viscosity decreased to 40 ± 1.8 mPa·s, indicating a risk of excessive binder flow (Table 5).

The performance improvements observed in modified asphalt binders are largely attributed to microstructural modifications that influence the binder’s internal network and rheological behavior. The observed rheological changes are attributed primarily to the physical reorganization of the binder rather than the formation of a new chemically bonded network. During high-temperature mixing, crumb-rubber particles absorb lighter aromatic fractions of the bitumen and partially swell, thereby increasing the effective volume of the dispersed rubber phase and restricting binder mobility. The cellulose fibers act as dispersed physical inclusions that may provide local mechanical reinforcement and further restrict binder flow. Consequently, the hybrid-modified binder develops a rougher, more heterogeneous, and mechanically constrained morphology containing modifier-rich domains. This physical restructuring is consistent with the observed increase in viscosity and G*/sinδ and the improvement in high-temperature deformation resistance. Furthermore, the absence of distinct new absorption bands in the FTIR-ATR spectra indicates that the observed structural changes were governed predominantly by physical interactions, partial rubber swelling, and mechanical constraint rather than the formation of new covalent bonds.

The reduction in penetration values clearly indicates that the binder has become stiffer and more resistant to mechanical loading, while the simultaneous increase in softening point reflects improved thermal stability and resistance to flow at high service temperatures. Previous studies consistently report that polymer- and rubber-based modifiers reduce penetration, increase softening point, and enhance durability under high-temperature conditions, ultimately improving long-term pavement performance [20]. The increase in the rutting parameter (G*/sinδ) measured using DSR further confirms the improved high-temperature deformation resistance of the hybrid-modified binder. This behavior is consistent with recent developments in sustainable binder modification, where waste-derived elastomeric materials are increasingly used to enhance high-temperature performance. The G*/sin δ parameter exceeding the Superpave threshold of 1 kPa demonstrates a significant improvement in rutting resistance. In the present study, the DSR critical temperature increased from approximately 64 °C for the base binder to 66 ± 1.5 °C for the unaged hybrid-modified binder and reached 71.2 ± 1.2 °C after ageing. These results indicate improved high-temperature deformation resistance within the investigated temperature range. Similarly, recent studies have reported that composite modification using waste-derived polymers such as recycled materials can provide performance levels comparable to or exceeding traditional SBS-modified binders, highlighting the growing potential of sustainable modifier systems [21,22]. The behavior of the binder after aging (RTFOT + PAV) also aligns well with established literature. Oxidative aging typically leads to structural hardening, reflected by increased softening point and reduced penetration and ductility values. In this study, the softening point increased by approximately 11 °C after aging, confirming the expected hardening effect due to oxidation [23,24,25]. Furthermore, the temperature corresponding to the G*/sin δ = 1 kPa criterion increased to 71.2 °C after PAV aging, indicating enhanced rutting resistance under long-term service conditions. Literature similarly reports that binders subjected to severe aging exhibit higher performance grades due to structural densification and oxidation-induced stiffness [26,27,28]. While aging generally increases stiffness, maintaining a balance between rutting resistance and cracking susceptibility is critical for long-term pavement durability.

Based on mechanisms reported in the literature, the observed rheological changes may be associated with the swelling of crumb-rubber particles and the physical restriction of binder mobility by the dispersed cellulose fibers. However, because cellulose-only and crumb-rubber-only control binders were not tested, the observed changes cannot be attributed quantitatively to either modifier, and a synergistic interaction cannot be confirmed. The results therefore represent the overall response of the selected hybrid-modified binder.

In order to contextualize these results within current engineering practice, a comparison with conventional and advanced binder systems is provided. When compared with conventional 50/70 penetration grade bitumen, which typically exhibits a high-temperature performance limit of approximately 64 °C, the modified binder in this study demonstrates a significantly improved performance level, reaching approximately 66–71 °C based on G*/sin δ criteria. This indicates a clear enhancement in rutting resistance. Furthermore, the obtained rheological properties approach those reported for polymer-modified binders such as SBS-modified systems (e.g., PG 70-22 or PG 76-22), suggesting that the proposed hybrid modification strategy can achieve comparable performance improvements using waste-derived materials.

These findings confirm that the developed binder not only meets standard specification limits but also provides performance levels that exceed conventional binders and align with advanced modified systems reported in the literature.

At intermediate temperatures, the long-term aged binder demonstrated adequate resistance to fatigue cracking. The G*·sin δ value reaching 5000 kPa at 24.3 °C confirms that the binder meets the Superpave fatigue criterion, which requires this value to remain below 5000 kPa at intermediate temperatures (e.g., 25 °C) [29]. Literature also indicates that certain modifiers, including small amounts of bio-based oils, can improve fatigue resistance by reducing stiffness at intermediate temperatures [30,31]. The ability of the modified binder in this study to maintain acceptable fatigue performance while enhancing rutting resistance reflects a well-balanced rheological profile.

Low-temperature performance is another critical factor in evaluating modified binders. The BBR results obtained at −12 °C indicate that the hybrid-modified binder satisfied the specified creep-stiffness and m-value limits at the tested temperature. Therefore, the combined modification did not adversely affect the low-temperature stress-relaxation response under the conditions evaluated in this study. At −12 °C, the hybrid-modified binder exhibited a creep stiffness (S) of 185 ± 8 MPa and an m-value of 0.326 ± 0.006. These values satisfied the Superpave limits of S ≤ 300 MPa and m ≥ 0.300, indicating acceptable low-temperature stress-relaxation performance at the investigated temperature. The acceptable BBR performance observed in this study confirms that the combined modification does not significantly compromise low-temperature behavior. One of the most notable side effects of modification is the increase in mixing viscosity. Brookfield viscosity measurements revealed a viscosity of approximately 2367 mPa·s at 150 °C, indicating that the modified binder is substantially more viscous than conventional bitumen and may present challenges during mixing and pumping operations. This increase in viscosity is widely reported for polymer- and rubber-modified binders and is associated with the formation of a denser internal network structure [32,33,34,35]. Based on viscosity temperature relationships, the optimal mixing and compaction temperature range for the modified binder was determined to be approximately 160–170 °C, which is consistent with recommended temperatures for polymer-modified systems reported in the literature [34,35]. However, excessively high temperatures (e.g., above 180 °C) may cause polymer degradation, accelerated binder aging, and increased emission of harmful fumes, negatively affecting both environmental and occupational safety [36,37]. Therefore, strict temperature control during production and application is essential to preserve the rheological advantages achieved through modification while minimizing environmental and operational risks.

Overall, these results indicate that the selected modifier proportions provided a reasonable balance between stiffness, elasticity, and workability under the investigated laboratory conditions.

### 3.4. Morphological Characteristics

SEM micrographs were examined at 2500× and 5000× magnifications. Modifier-rich domains were distributed across the examined surfaces; however, localized agglomerated regions indicated that completely uniform modifier dispersion was not achieved under the applied mixing conditions. The SEM micrographs show that the hybrid modification transformed the relatively smooth and homogeneous surface of the base bitumen into a rougher and more heterogeneous morphology containing modifier-rich domains. However, clearly distinguishable cellulose fibers were not observed on the examined surfaces. This may be attributed to the coating and partial embedding of the fibers within the bituminous matrix during high-temperature mixing. In addition, the limited field of view and surface-sensitive nature of SEM imaging may have prevented exposed fiber segments from being captured. Therefore, the micrographs demonstrate increased morphological heterogeneity but do not directly confirm the presence of a continuous cellulose-fiber network. Dispersion and agglomeration were evaluated qualitatively, and no quantitative image analysis was performed. Accordingly, the SEM observations suggest possible local physical reinforcement but do not conclusively demonstrate a continuous reinforcement network. The morphological characteristics of the base and hybrid-modified binders were initially examined using SEM, as shown in Figure 1.

Figure 2 compares the normalized FTIR spectra of the original 50/70 bitumen, waste-derived cellulose fibers, crumb rubber, and the hybrid-modified binder. The principal absorption bands and their corresponding tentative assignments are summarized in Table 6. The spectra of the individual modifiers were used to support the tentative assignment of the characteristic bands observed in the fingerprint region of the hybrid-modified binder. The dominant bands at approximately 2920 and 2851 cm^−1^ were assigned to the asymmetric and symmetric stretching vibrations of aliphatic CH_2_ groups, respectively. Additional bands observed at approximately 1458 and 1375–1376 cm^−1^ were associated with CH_2_ and CH_3_ deformation vibrations. The band located near 1600–1604 cm^−1^ was attributed to aromatic C=C stretching, while the absorption band at approximately 723 cm^−1^ corresponded to the rocking vibration of long-chain methylene groups.

The principal absorption bands of the base binder remained present in the hybrid-modified binder without substantial shifts in their positions. This similarity indicates that the fundamental chemical structure of the bitumen was preserved following modification. Nevertheless, relative spectral changes were observed in the fingerprint region, particularly around 1158, 1011–1030, 954, 863, and 809 cm^−1^. The variations near 1158 and 1011–1030 cm^−1^ may be associated with C–O and C–O–C vibrations originating from the cellulose component, although overlap with bitumen-related bands, including the sulfoxide region, should be considered. Changes at approximately 954, 863, and 809 cm^−1^ may reflect the contribution of crumb-rubber-related and aromatic out-of-plane C–H vibrations.

The combined SEM and FTIR analyses provide complementary evidence regarding the modification mechanism and structural development of the hybrid-modified asphalt binder. As illustrated in Figure 1, the unmodified 50/70 penetration-grade bitumen exhibited a relatively smooth, continuous, and homogeneous surface at both 2500× and 5000× magnifications. In contrast, the hybrid-modified binder displayed a substantially rougher, more heterogeneous, and structurally complex morphology. Irregular modifier-rich domains of different sizes were distributed throughout the bituminous matrix, and several particles appeared to be partially embedded in or surrounded by the continuous binder phase. These features indicate physical adhesion and mechanical interlocking between the modifiers and the bitumen. Localized agglomerated regions were also observed, demonstrating that completely uniform modifier dispersion was not achieved under the applied mixing conditions. Nevertheless, the resulting morphological complexity may restrict molecular mobility and strengthen the internal structure of the binder, thereby contributing to the observed increases in viscosity, stiffness, softening point, and resistance to permanent deformation.

Overall, the SEM and FTIR findings are mutually consistent and support the formation of a physically reinforced binder system without fundamental alteration of the chemical framework of the base bitumen. This combined microstructural and physicochemical evidence provides a plausible explanation for the improved rheological response, thermal stability, and high-temperature deformation resistance of the hybrid-modified binder.

## 4. Conclusions

This study evaluated the rheological, workability, morphological, and chemical characteristics of a 50/70 penetration-grade asphalt binder hybrid-modified with waste-derived cellulose fibers and crumb rubber. The hybrid modification increased binder stiffness, softening point, and the G*/sinδ parameter, indicating improved high-temperature stability and resistance to permanent deformation. The modified binder reached an upper service temperature of approximately 66–71 °C. However, the increase in viscosity indicates that careful control of mixing and compaction temperatures is required to maintain adequate workability during production and application.

Overall, the combined rheological, SEM, and FTIR findings indicate that waste-derived cellulose fibers and crumb rubber can be used to produce a more structurally complex and deformation-resistant asphalt binder without fundamentally altering the chemical structure of the base bitumen. The proposed approach also promotes the valorization of textile and tire wastes and may reduce dependence on virgin modifier materials, thereby supporting circular economy principles in pavement engineering. Although related rubber- and fiber-modified asphalt systems have previously been investigated, the contribution of the present study is the integrated binder-scale evaluation of recycled cotton-derived cellulose fibers and crumb rubber using rheological, ageing, low-temperature, morphological, and spectroscopic analyses. The study does not claim the first-ever application of these modifiers.

Several limitations should nevertheless be acknowledged. Because BBR testing was conducted only at −12 °C, the results confirm compliance only with the selected PG −22 grade and do not establish the complete low-temperature performance-grade boundary. Future studies should therefore include additional tests at lower temperatures. The investigation was conducted under controlled laboratory conditions and may not fully represent field-scale production, environmental exposure, traffic loading, storage stability, and long-term pavement performance. Furthermore, the study examined a selected hybrid formulation without conducting a comprehensive parametric optimization or direct comparison with corresponding single-modifier binders. Therefore, the individual contributions of cellulose fibers and crumb rubber and any potential synergistic effects cannot yet be quantified conclusively. Future studies should include single-modifier control groups, optimization of modifier proportions and mixing conditions, storage-stability assessment, mixture-level mechanical testing, field-scale validation, long-term performance monitoring, and detailed life-cycle and economic analyses.

## Figures and Tables

**Figure 1:**
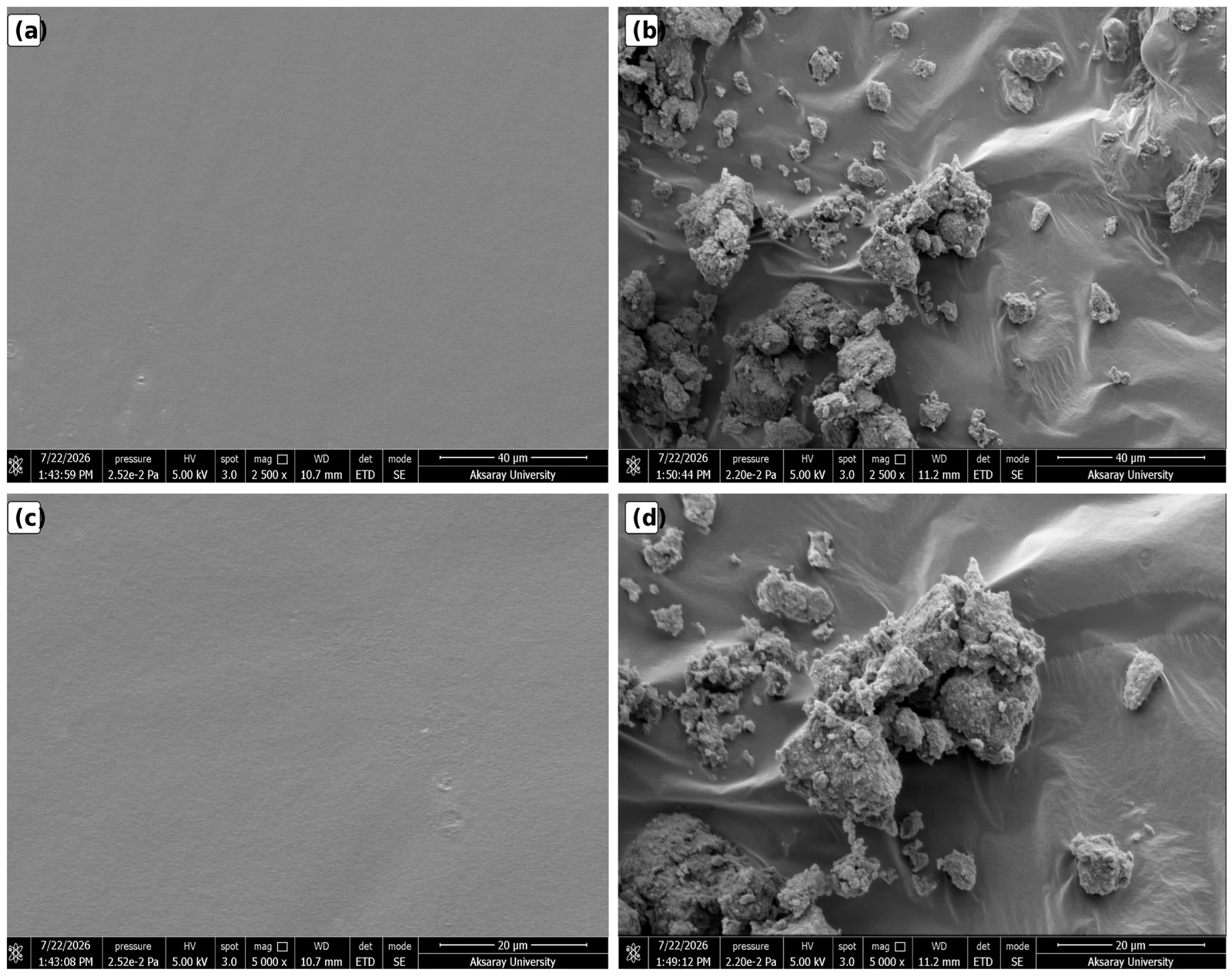
SEM micrographs of (**a**,**c**) the original 50/70 base binder and (**b**,**d**) the hybrid-modified binder at (**a**,**b**) 2500× and (**c**,**d**) 5000× magnifications. (The original instrument-generated scale bars are shown in each micrograph).

**Figure 2:**
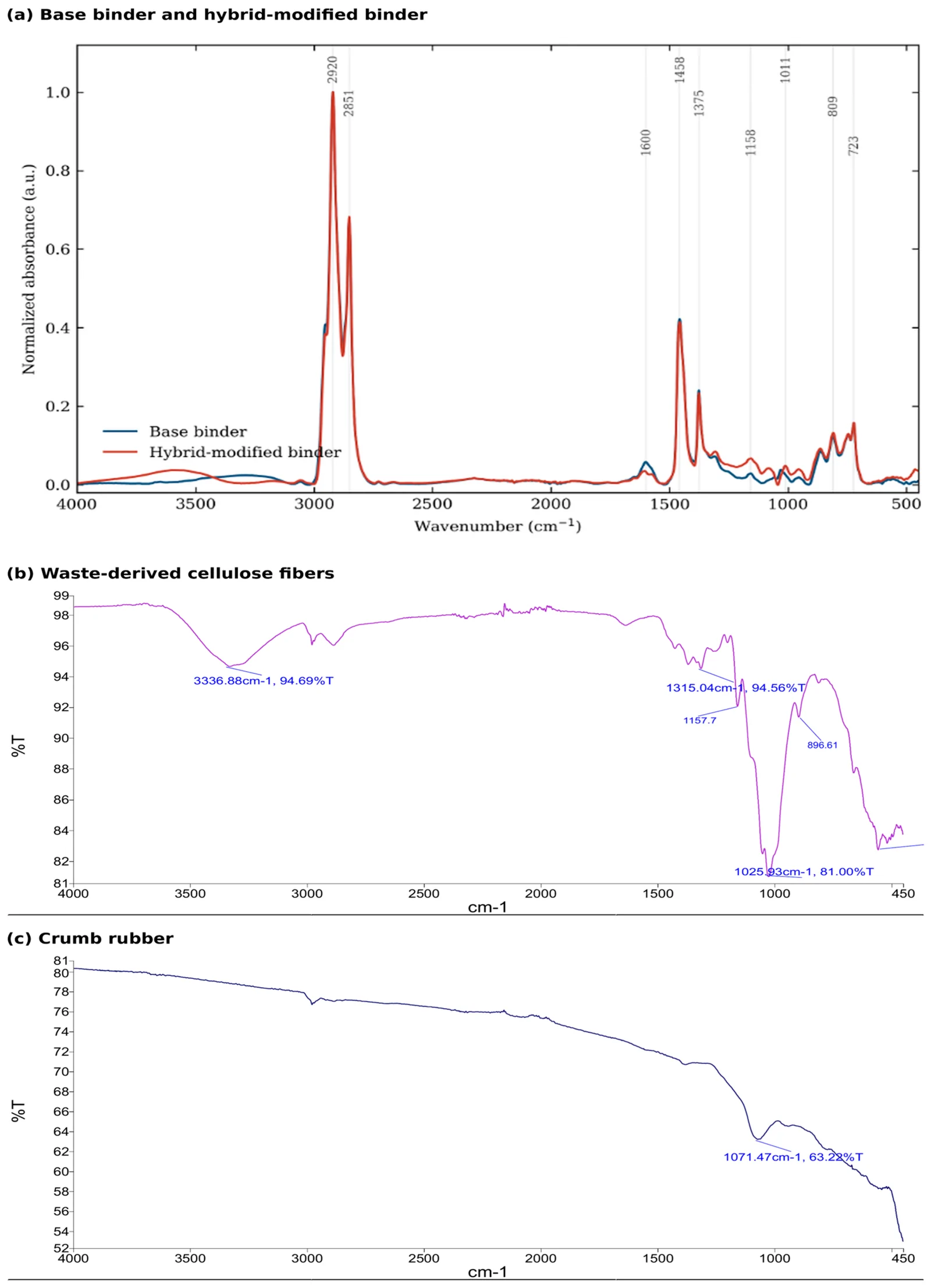
FTIR-ATR spectra of (**a**) the base binder and hybrid-modified binder, (**b**) waste-derived cellulose fibers, and (**c**) crumb rubber. The individual modifier spectra are presented in transmittance mode, whereas the binder spectra are presented as normalized absorbance.

**Table 1:** Characteristics of the raw materials used in the study.

Material	Source and Form	Preparation/Production Method	Main Characteristics	Dosage
Base bitumen	50/70 penetration-grade petroleum bitumen obtained from [Aksaray, Turkiye]	Used as received	Brookfield viscosity at 135 °C: 320 mPa·s; G* at 60 °C: 1.20 kPa; phase angle at 60 °C: 86°	88 wt%
Waste-derived cellulose fibers	Post-consumer cotton-based textile waste obtained from [Aksaray, Turkiye]	Cleaning, shredding, alkali treatment, and controlled drying		5 wt%
Crumb rubber	End-of-life vehicle tires obtained from [Aksaray, Turkiye]	Ambient mechanical grinding		7 wt%

Note: Modifier contents are expressed as percentages of the total binder mass.

**Table 2:** Primary Tests and Measured Parameters.

Test	Standard	Equipment Manufacturer and Model	Test Conditions	Measured Parameter
Penetration	TS EN 1426	UTEST, Ankara, TURKİYE	25 °C; 100 g load; 5 s loading time	Penetration depth (0.1 mm)
Softening point	TS EN 1427	UTEST, UTB-0210, Ankara, TURKİYE	Ring-and-ball method; heating rate of 5 °C/min	Softening temperature (°C)
Brookfield viscosity	ASTM D4402/D4402M	AMETEK Brookfield, DV2T, Middleboro, MA, USA	150–180 °C; 20 rpm	Rotational viscosity (mPa·s)
DSR	TS EN 14770; AASHTO T315	Anton Paar GmbH, SmartPave 102e, Graz, Austria	25 mm parallel plates; 1 mm gap; temperature sweep from 52 to 82 °C; angular frequency of 10 rad/s	Complex shear modulus (G*), phase angle (δ), and rutting parameter (G*/sinδ)
RTFOT	TS EN 12607-1; AASHTO T240	UTEST, UTAS-0140, Ankara, TURKİYE	163 °C for 85 min; airflow rate of 4000 ± 200 mL/min	Short-term ageing characteristics
PAV	TS EN 14769; AASHTO R28	Applied Test Systems (ATS), PAV4, Butler, PA, USA	100 °C; 2.1 ± 0.1 MPa; 20 h	Long-term ageing characteristics
BBR	TS EN 14771; AASHTO T313; ASTM D6648	Applied Test Systems (ATS), BBR3, Butler, PA, USA	−12 °C; 980 ± 50 mN constant load; 60 s loading time	Creep stiffness (S) and m-value
SEM	—	FEI Company, Quanta FEG 250, Hillsboro, OR, USA	kV; magnifications of 2500× and 5000×	Surface morphology, modifier dispersion, adhesion, and agglomeration
FTIR-ATR	—	PerkinElmer, Spectrum Two, Waltham, MA, USA	Spectral range of 4000–400 cm^−1^; resolution of 4 cm^−1^; scans per spectrum	Functional groups and physicochemical interactions

**Table 3:** Fundamental Properties of the Orginal Bitumen and Modified Binder.

Property	Unit	Original 50/70 Bitumen	Hybrid-Modified Binder (Mean ± SD)	Interpretation
Penetration at 25 °C (100 g, 5 s)	0.1 mm	50–70	55 ± 2	The modified binder remained within the specified penetration range
Softening point	°C	46–54	53 ± 1	The modified binder approached the upper specification limit
Brookfield viscosity	mPa·s	320 at 135 °C	2367 ± 45 at 150 °C	Modification substantially increased viscosity
DSR critical temperature at G*/sinδ ≥ 1 kPa	°C	64	66 ± 1.5	Modification increased the upper service temperature
Mass change after RTFOT	%	≤0.50	0.32 ± 0.05	The modified binder satisfied the specified ageing limit
Retained penetration after RTFOT	%	≥50	58 ± 3	The modified binder retained acceptable flexibility after ageing

**Table 4:** Aging Resistance and Rheological Parameters (RTFOT & PAV).

Parameter	Unit	Result (Mean ± SD)	Interpretation
Softening point increase after ageing	°C	+11 ± 0.8	Ageing-induced stiffening
DSR critical temperature at G*/sinδ = 1 kPa	°C	71.2 ± 1.2	Increased high-temperature stiffness
DSR critical temperature at G*·sinδ = 5000 kPa	°C	24.3 ± 0.9	Fatigue-related performance
Creep stiffness, S, at −12 °C	MPa	185 ± 8	Criterion satisfied
m-value at −12 °C	—	0.326 ± 0.006	Criterion satisfied

Note. RTFOT: Rolling Thin Film Oven Test; PAV: Pressure Aging Vessel. BBR: Bending Beam Rheometer. DSR: Dynamic Shear Rheometer. Thresholds follow Superpave specifications.

**Table 5:** Brookfield Viscosity and Processing Window.

Temperature (°C)	Viscosity (mPa·s)	Torque (%)	Spindle Speed (rpm)	Qualitative Evaluation
150	2367 ± 45	94.7 ± 1.2	20	High viscosity: higher mixing temp required
160	1649 ± 35	67.2 ± 1.0	20	Suitable processing range
170	68.8 ± 2.5	2.8 ± 0.2	20	Easier workability
180	40 ± 1.8	1.6 ± 0.1	20	Risk of excessive flow

Note. All measurements were taken using a Brookfield rotational viscometer with constant spindle speed (20 rpm). Elevated torque values at lower temperatures indicate high binder stiffness and mixing limitations.

**Table 6:** Principal FTIR absorption bands identified in the base and hybrid-modified asphalt binders and their corresponding assignments.

Wavenumber (cm^−1^)	Tentative Vibrational Assignment	Interpretation
2920	Asymmetric aliphatic CH_2_ stretching	Hydrocarbon chains originating from the bitumen and crumb rubber
2851	Symmetric aliphatic CH_2_ stretching	Long-chain aliphatic structures
1600–1604	Aromatic C=C stretching	Aromatic components of the bituminous binder
1458	CH_2_ scissoring and CH_3_ deformation	Aliphatic hydrocarbon structure
1375–1376	Symmetric CH_3_ bending	Branched aliphatic groups
1158	C–O–C and C–O vibrations	Possible contribution from the cellulose component
1011–1030	C–O and/or S=O vibrations	Overlapping contributions from cellulose and bitumen-related sulfoxide groups
954	Out-of-plane C–H vibration	Possible contribution from the crumb rubber structure
862–863	Aromatic out-of-plane C–H vibration	Aromatic components of bitumen and crumb rubber
809	Out-of-plane C–H vibration	Aromatic and rubber-related structures
723	Long-chain CH_2_ rocking vibration	Long hydrocarbon chains present in the bituminous binder

Note: The assignments are tentative because characteristic bands originating from bitumen, cellulose, and crumb rubber may overlap within the FTIR fingerprint region.

## Data Availability

Data are contained within the article. The data supporting the findings of this study are available from the corresponding author upon reasonable request.
